# DPARD: rationale, design and initial results from the Dutch national diabetes registry

**DOI:** 10.1186/s12902-021-00782-x

**Published:** 2021-06-16

**Authors:** Jessica C. G. Bak, Dick Mul, Erik H. Serné, Harold W. de Valk, Theo C. J. Sas, Petronella H. Geelhoed-Duijvestijn, Mark H. H. Kramer, Max Nieuwdorp, Carianne L. Verheugt

**Affiliations:** 1 Department of Vascular Medicine, Amsterdam University Medical Centres, location AMC, Meibergdreef 9, 1105 AZ Amsterdam, The Netherlands; 2 Dutch Institute for Clinical Auditing, Leiden, The Netherlands; 3 Diabeter, Centre for paediatric and adult diabetes care and research, Rotterdam, The Netherlands; 4 Department of Internal Medicine, University Medical Centre Utrecht, Utrecht, The Netherlands; 5 Department of Pediatric Endocrinology, Erasmus Medical Centre, Rotterdam, The Netherlands; 6 Department of Internal Medicine, Haaglanden Medical Centre, The Hague, The Netherlands

**Keywords:** National diabetes registry, Diabetes mellitus, Epidemiology, Quality of care, Outpatient diabetes care

## Abstract

**Background:**

Treatment of diabetes mellitus has majorly improved over the past century, however, the disease burden is high and its prevalence still expanding. Further insight in the diabetes population is imperative to improve the quality of diabetes care by enhancement of knowledge-based diabetes management strategies. To this end, in 2017 a Dutch nationwide consortium of diabetologists, paediatric endocrinologists, and diabetes patients has founded a national outpatient diabetes care registry named Dutch Pediatric and Adult Registry of Diabetes (DPARD). We aim to describe the implementation of DPARD and to provide an overview of the characteristics of patients included during the first 2 years.

**Methods:**

For the DPARD cohort with long-term follow-up of observational nature, hospital data are gathered directly from electronic health records and securely transferred and stored. DPARD provides weekly updated clinical information on the diabetes population care on a hospital-level benchmarked against the national average.

**Results:**

Between November 2017 and January 2020, 20,857 patients were included from 8 (11%) Dutch hospitals with a level of care distribution representative of all diabetic outpatients in the Netherlands. Among patients with known diabetes type, 41% had type 1 diabetes, 51% type 2 diabetes, and 8% had diabetes due to other causes. Characteristics of the total patient population were similar to patients with unknown diabetes classification. HbA1c levels decreased over the years, while BMI levels showed an increase over time.

**Conclusions:**

The national DPARD registry aims to facilitate investigation of prevalence and long-term outcomes of Dutch outpatients with diabetes mellitus and their treatment, thus allowing for quality improvement of diabetes care as well as allowing for comparison of diabetes care on an international level.

## Background

In the past four decades, the global prevalence of diabetes mellitus in adults has nearly doubled due to ageing and increasing rates of overweight and obesity, with a current estimated number of 463 million adults worldwide [1]. In the Netherlands, the current approximated national prevalence of diabetes mellitus at all ages is 6,9%, with an estimated number of 1,2 million patients, a number expected to rise to 1,4 million by 2040 [2]. These epidemic proportions of diabetes mellitus go together with high rates of comorbidity, microvascular and macrovascular complications, a lower life expectancy, and a decrease in reported quality of life [3–5]. The burden of diabetes mellitus on national health care services is equally high, and is expected to rise in future decades as the diabetes mellitus population will further age and expand. Achieving treatment targets in diabetes care reduces microvascular complications and cardiovascular events [6]. It has been shown that in a substantial part of the diabetes population, treatment goals regarding blood pressure, glycemic control and lipid regulation were not achieved, [7] while intensive therapy with close monitoring has shown to reduce microvascular complications [8].

In order to maintain high quality, proper allocation, and coverage of health care services for patients with diabetes mellitus, a clear overview as well as in-depth clinical information on this population is vital [9]. National disease-specific quality registers are known to promote health care quality and efficacy by evaluating and benchmarking quality of care, and by identifying areas in need of improvement [10, 11]. To this end, several countries around the world have succesfully initiated national registries on diabetes mellitus [12, 13]. In the Netherlands, data on the prevalence and outcomes of the diabetes population have up until now primarily been derived from general practice databases, or from declaration databases in children [14, 15]. While the importance of these outcomes for the diabetes population treated by the general practitioner is clear, these outcomes pertain less to Dutch outpatient diabetes population, whose disease pattern, resources and needs are quite different. This essential gap in in-depth, systematic, and long-term clinical information on Dutch diabetes outpatients has led to the launch of the Dutch Pediatric and Adult Registry of Diabetes (DPARD) in 2017 by the BIDON foundation, a Dutch nationwide consortium of paediatric endocrinologists, diabetologists, and diabetes patients.

DPARD aims to provide insight in the characteristics of the Dutch diabetes outpatient population and enhance knowledge-based diabetes management strategies, aiming to reduce disease burden and improve quality of diabetes care, as other databases have proven to accomplish [16, 17]. Simultaneously, DPARD provides information on clinical outcomes and healthcare reimbursement to healthcare institutions and their professionals, alongside with benchmark information to increase transparency of the quality of diabetes care in all ages across the Netherlands.

This study primarily aims to describe the rationale and design of the DPARD national registry and, additionally, to yield its first results.

## Methods

### Registry population and initiation

All patients with diabetes mellitus treated in outpatient clinics in secondary and tertiary care, both children and adults, are eligible for inclusion in DPARD [18]. Exclusion criteria are gestational diabetes and diabetes mellitus merely requiring consultation during hospitalization while normally receiving treatment for diabetes mellitus in primary care. Primary care may come in focus in the future. Patients treated at private outpatient clinics are also included in DPARD. The number of patients with diabetes mellitus in the Netherlands is estimated on 1.2 million, approximately 185,000 of whom are treated in outpatient clinics and 810,000 patients only treated in primary care [2]. In 2017, delegates of The Netherlands Association of Internal Medicine (NIV), The Dutch Association for Paediatrics (NVK), and the Dutch federation of diabetes patients (DVN) united in the BIDON foundation have initiated DPARD. Since 2018, the Dutch Institute for Clinical Auditing (DICA), known for the facilitation of nationwide audits in a uniform format, is responsible for the facilitation of DPARD [19].

### Data collection and validation

The DPARD dataset includes 119 parameters grouped into four categories: 1) patient and disease characteristics, i.e. date of birth, ethnicity, date of diagnosis, diabetes classification; 2) process parameters used in the diagnostic process and follow-up, such as clinical examinations and laboratory parameters; 3) complications and comorbidity; 4) treatment comprising glucose-lowering therapy, treatment of comorbidities, and continuous glucose monitoring. The selection of relevant variables for the DPARD data set is based on the standard set of International Consortium of Health Outcome Measurement (ICHOM), [20] as well as national and international diabetes guidelines [21, 22]. Diagnostic procedures of patients with diabetes mellitus in the Netherlands are based on the WHO classification. Antibody status was provided when available in the DPARD database, however in most electronic health records antibody status was not recorded discretely and therefore not available. Diabetes mellitus is diagnosed according to American Diabetes Association (ADA) and International Society for Pediatric and Adolescent Diabetes (ISPAD) guidelines [23, 24]. Diabetes classification is typically based on characteristics at presentation according to previous mentioned guidelines. In patients with type 1 diabetes mellitus, antibody testing (GAD, IA2, IAA, and/or ZnT8 antibodies) is performed in every newly diagnosed patient. In children, antibody-negative patients are further studied to assess monogenetic causes of diabetes, with both (family) history and (low) insulin need as additional informative items, and genetic analysis in possible cases of monogenic diabetes. In adults family history and patient characteristics can give indication to genetic analysis for monogenetic causes. In hybrid forms, clinical characteristics and diabetes antibodies will also guide to diagnosis. The initial diabetes classification in DPARD is based on the WHO classification [25]. Dataset content is evaluated and modified on a yearly basis. Data dictionaries are available online [26]. Data are collected directly from local hospitals’ electronic health records by electronic health record specialists, who selected the DPARD variables from the records of included patients, and entered these variables into so-called batch files or batches. Batches are series of data processed as a group. Batches are electronically delivered to Medical Research Data Management (MRDM), [27] a trusted third party responsible for securely processing, storing and encrypting data. To ensure cyber security during collection and transmission of data, data collection is done by qualified persons from the local hospitals in their secure IT environment and data is transmitted via the secured upload facility of MRDM. Data are encrypted directly after entry, preventing data from being traced back to individual patients. Unique non-traceable identification numbers are assigned to every patient, allowing follow-up over time.

All methods used in this study were carried out in accordance with relevant guidelines and regulations. MRDM is compliant with Dutch and European privacy laws, [28] and is NEN 7510:2011 and ISO certified [29]. Participating hospitals remain ownership of their data and are responsible for the completeness and correctness of their data. In accordance to the Dutch and European Privacy Protection Laws, no ethical approval or informed consent was required for this study. A detailed description of the data entry and verification process is provided by Hoeijmakers et al. [30] In short, data validation takes place via internal and external validation. Internal validation occurs by validation of submitted batches by MRDM for missing data and unrealistic answers. Validation reports are generated summarizing potential errors. ICT workers from health care institutions verify and correct their data and provide an adjusted batch file. External validation is performed every 3 years by an independent monitoring committee comparing source data in electronic health records with a random data sample from the registry. During this process, the completeness and ascertainment of patient inclusion is checked, as well as accuracy and completeness of key variables. Feedback information is provided to participating hospitals via weekly updated online reports by use of the Codman dashboards [19]. Results for structure, process, and outcome indicators from multiple hospitals are graphically benchmarked against the national average by using funnel plots with 95% confidence intervals around the national average. Individual hospitals are anonymized in these reports.

### Statistical analysis and definitions

For this cohort study with long-term follow-up of observational nature, we included all patients registered in DPARD visiting a Dutch outpatient clinic between January 1, 2016, and January 1, 2020, thus allowing for inclusion of patients from the year in which nearly all Dutch hospitals had implemented electronic patient records in their daily practice. In DPARD, patients are followed-up every year during visits to the diabetes outpatient clinic until referral back to primary care, curation, or death. Patients with missing or unrealistic dates of last outpatient clinic visit were excluded. Diabetes type was based on the clinical classification entered in electronic health records by medical professionals. BMI was calculated as weight in kilograms divided by height square in meters, using a cut-off value of 25 kg/m^2^ for overweight and 30 kg/m^2^ for obesity in adults. Misclassification was defined as classifying a patient in an inaccurate diagnosis category. In misdiagnosis, an incorrect diagnosis was allocated. Miscoding was defined as the recording of a correct diagnosis into an incorrect diagnose code, for example ICD-10 coding [31].

Descriptive statistics were used to assess patient, disease- and treatment characteristics. Due to non-parametrical distribution of our data, medians were used for descriptives. Means were also calculated for comparison with other national databases. Statistical analyses were performed using SPSS (IBM SPSS Statistics for Windows, version 26.0) and R (RStudio, version 1.2.5019). Rates of missing data were shown in tables or mentioned in the results. Missing data were included in all analyses unless mentioned otherwise.

## Results

Between November 2017 and January 2020, a total of 20,857 patients were included in DPARD, 18,714 patients of whom have visited the outpatient clinic at least once during those years. A total of 17,784 adults (95%) and 930 children (5%) were used for analysis. During this period, 8 medical centres have transferred data (two tertiary care centres, five secondary hospitals, and one independent diabetes treatment centre) constituting of approximately 11% of all Dutch hospitals with a level of care distribution that is representative of the total diabetic outpatient population in the Netherlands. In addition, 18 hospitals are in the connection process to upload data plus another 18 hospitals that have shown interest to participate, resulting in a 44% coverage of all Dutch hospitals involved in DPARD to a certain degree.

Table 1 shows the characteristics of all patients included in DPARD by diabetes type. In total, 4175 (22%) patients were diagnosed with type 1 diabetes mellitus, 5158 patients (28%) with type 2 diabetes mellitus; 822 patients (4%) had secondary diabetes mellitus such as drug-induced diabetes mellitus or other causes of diabetes mellitus including genetic syndromes, and of 8559 patients (46%) the diabetes classification was not conveyed by the hospitals. Among patients with known classification, 41% had type 1 diabetes mellitus, 51% type 2 diabetes mellitus and 8% were diagnosed with secondary or other causes of diabetes mellitus. The median age in type 1 was 30.0 years (mean age 36.5 years, range 5.0–95.0 years) whereas the median age in type 2 diabetes was 67.0 years (mean age 65.9 years). Concomitantly, the median diabetes duration was longer in type 2 diabetes patients (18 years) than in patients with type 1 (15 years). In patients with secondary or other causes of diabetes mellitus, the median age was similar to type 1 diabetes patients (34.0 years) and diabetes duration was the shortest of all diabetes types (3 years). Gender distribution was similar among type 1 and type 2 diabetes patients, yet the category of secondary or other causes of diabetes mellitus comprised predominately of women.

**Table 1:** Baseline characteristics of patients included in the Dutch Pediatric and Adult Registry of Diabetes (DPARD)

	Total	DM type 1	DM type 2	DM other/secondary	Unclassified
	(*n* = 18,714)	(*n* = 4175)	(*n* = 5158)	(*n* = 822)	(*n* = 8559)
Age (years)	56.0 (5.0–98.0)	30.0 (5.0–95.0)	67.0 (14.0–98.0)	34.0 (5.0–82.0)	56.0 (5.0–97.0)
Male sex (%)	52.1	54.8	58.1	22.9	50.1
Diabetes duration (years)	15.0 (1.0–81.0)	15.0 (1.0–81.0)	18.0 (1.0–63.0)	3.0 (1.0–49.0)	15.0 (1.0–70.0)
Smoker (%)	10.0	7.3	12.6	4.5	10.2
Non-smoker (%)	51.6	35.7	62.5	15.8	56.3
Unknown (%)	38.4	57.0	24.9	79.7	33.5
BMI (kg/m^2^)	27.7 (12.9–50.0)	25.3 (16.2–44.1)	29.4 (15.1–49.8)	28.2 (13.8–40.7)	27.6 (12.9–50.0)
< 20 (%)	3.1	2.5	0.6	1.3	5.0
20–24 (%)	17.3	16.4	10.4	10.3	22.7
25–29 (%)	24.1	16.5	26.3	15.8	27.2
≥ 30 (%)	23.4	4.6	30.1	12.1	29.7
Unknown (%)	32.1	60.0	32.6	60.5	15.4
Systolic blood pressure (mmHg)	133.0 (65–250.0)	130.0 (65–215.0)	136.0 (71.0–250.0)	120.0 (84.0–208.0)	130.0 (72–200)
Diastolic blood pressure (mmHg)	76.0 (21.0–125.0)	75.0 (45.0–118.0)	77.0 (21.0–125.0)	72.0 (50.0–124.0)	80.0 (50.0–114.0)
eGFR (ml/min/1.73m^2^)	77.0 (2.0–100.0)	78.0 (4.0–100.0)	66.0 (4.0–100.0)	83.5 (31.0–100.0)	82.0 (2.0–100.0)
Urinary albumin (mg/l)	11.0 (0.0–9520.0)	7.0 (0.0–3903.0)	18.0 (0.0–9520.0)	15.0 (1.0–1091.0)	10.0 (2.0–5738.0)
HDL- cholesterol (mmol/l)	1.2 (0.2–9.9)	1.5 (0.4–4.0)	1.1 (0.2–4.7)	1.1 (0.5–3.7)	1.3 (0.2–9.9)
LDL-cholesterol (mmol/l)	2.4 (0.1–10.1)	2.6 (0.4–6.0)	2.3 (0.1–9.5)	2.6 (0.8–5.8)	2.3 (0.1–10.1)
HbA1c (mmol/mol)	60.0 (25.0–150.0)	61.0 (26.0–149.0)	63.0 (27.0–144.0)	44.0 (25.0–129.0)	59.0 (25.0–150.0)
< 53 (%)	25.6	20.6	18.1	48.3	30.4
< 64 (%)	55.2	56.2	46.0	59.6	59.9
< 86 (%)	85.3	87.7	79.8	71.5	88.9
HbA1c (%)	7.6 (4.4–15.9)	7.7 (4.5–15.8)	7.9 (4.6–15.3)	6.2 (4.4–14.0)	7.5 (4.4–15.9)

Numbers are stated as median (range) or percentage (%)

Adult type 2 patients had markedly higher BMI levels (29.4 kg/m^2^ and 30.1% obese) and urinary albumin (18.0 mg/l) compared to adult type 1 patients (BMI 25.3 kg/m^2^ and 5.3% obese patients, urinary albumin 7.0 mg/l). Median HbA1c was higher in type 2 diabetes (63 mmol/mol, 7.9%) than type 1 diabetes (61 mmol/mol, 7.7%). Patients with other or secondary types of diabetes mellitus showed lower blood pressure, higher eGFR, lower urinary albumin, higher LDL-cholesterol and lower HbA1c compared to type 1 and type 2 diabetes patients. Patients in whom the diabetes classification was not reported, had characteristics similar to those of the entire DPARD population.

Table 2 shows the characteristics of adult and paediatric patients included in DPARD. Comparable gender distribution was seen between adults and children. Most paediatric patients had a BMI under 20 kg/m^2^. The majority of adult patients had a BMI above 25 kg/m^2^. Among adults, type 2 diabetes was most common (54% of patients with known diabetes classification), whereas type 1 diabetes was most prevalent among children (85% of known diabetes classification). Testing on both islet cell (ICA), anti-glutamic acid decarboxylase (GAD) and islet antigen 2 (IA2) antibodies was known to be performed in 16.6% of children and 1.4% of the adults. In 60% of the children (90.9% of the children with type 1) and 8.5% of the adults (38.2% of the adults with type 1) at least one of the autoantibodies ICA, GAD or IA2 was assessed. MODY screening was known to be performed in 4.6% of the children and 0.6% of the adults. Urinary albumin was higher in adult than in paediatric patients (10.0 versus 5.0 mg/l). Median HbA1c levels were similar in both groups with 59 mmol/mol (7.5%) in adults and 60 mmol/mol (7.6%) in children. Median HbA1c levels in type 1 diabetes were also comparable with levels of 61 mmol/mol (7.7%) in adults and 60 mmol/mol (7.6%) in children. In contrast, paediatric subjects with type 2 diabetes had lower median HbA1c levels (35 mmol/mol, 5.4%) compared to adults (63 mmol/mol, 7.9%) with type 2.

**Table 2:** Characteristics of adult and paediatric outpatients included in Dutch Pediatric and Adult Registry of Diabetes

	Total	Adults	Children
	(*n* = 18,714)	(*n* = 17,784)	(*n* = 930)
Age (years)	56.0 (5.0–98.0)	57.0 (18.0–98.0)	14.0 (5.0–17.0)
Male sex (%)	52.1	52.3	48.8
Diabetes duration (years)	15.0 (1.0–81.0)	17.0 (1.0–81.0)	6.0 (2.0–16.0)
Diabetes type			
Type 1 (%)	22.3	20.3	60.4
Type 2 (%)	27.6	29.0	0.2
Other/secondary (%)	4.4	4.1	10.2
Unknown (%)	45.7	46.6	29.2
Smoking status			
Smoker (%)	10.0	10.5	0.2
Non-smoker (%)	51.6	53.5	15.1
Unknown (%)	38.4	36.0	84.7
BMI (kg/m^2^)	27.7 (12.9–50.0)	27.9 (12.9–50.0)	19.4 (13.6–34.0)
< 20 (%)	3.1	2.3	17.4
20–24 (%)	17.3	17.8	9.6
25–29 (%)	24.1	25.2	2.0
≥ 30 (%)	23.4	24.6	0.1
Unknown (%)	32.1	30.1	70.9
Systolic blood pressure (mmHg)	133.0 (65.0–250.0)	133.0 (65.0–250.0)	NA
Diastolic blood pressure (mmHg)	76.0 (21.0–125.0)	76.0 (21–125.0)	NA
eGFR (ml/min/1.73m^2^)	77.0 (2.0–100.0)	77.0 (2.0–100.0)	NA
Urinary albumin (mg/l)	11.0 (0.0–9520.0)	11.0 (0.0–9520.0)	5.0 (3.0–292.0)
HDL cholesterol (mmol/l)	1.2 (0.2–9.9)	1.2 (0.2–9.9)	1.6 (0.7–3.1)
LDL cholesterol (mmol/l)	2.4 (0.1–10.1)	2.4 (0.1–10.1)	2.4 (0.7–5.0)
HbA1c (mmol/mol)	60.0 (25.0–150.0)	60.0 (25.0–150.0)	59.0 (32.0–130.0)
HbA1c (%)	7.6 (4.4–15.9)	7.6 (4.4–15.9)	7.5 (5.1–14.0)

Numbers are stated as median (range) or percentage (%)

**Fig. 1:**
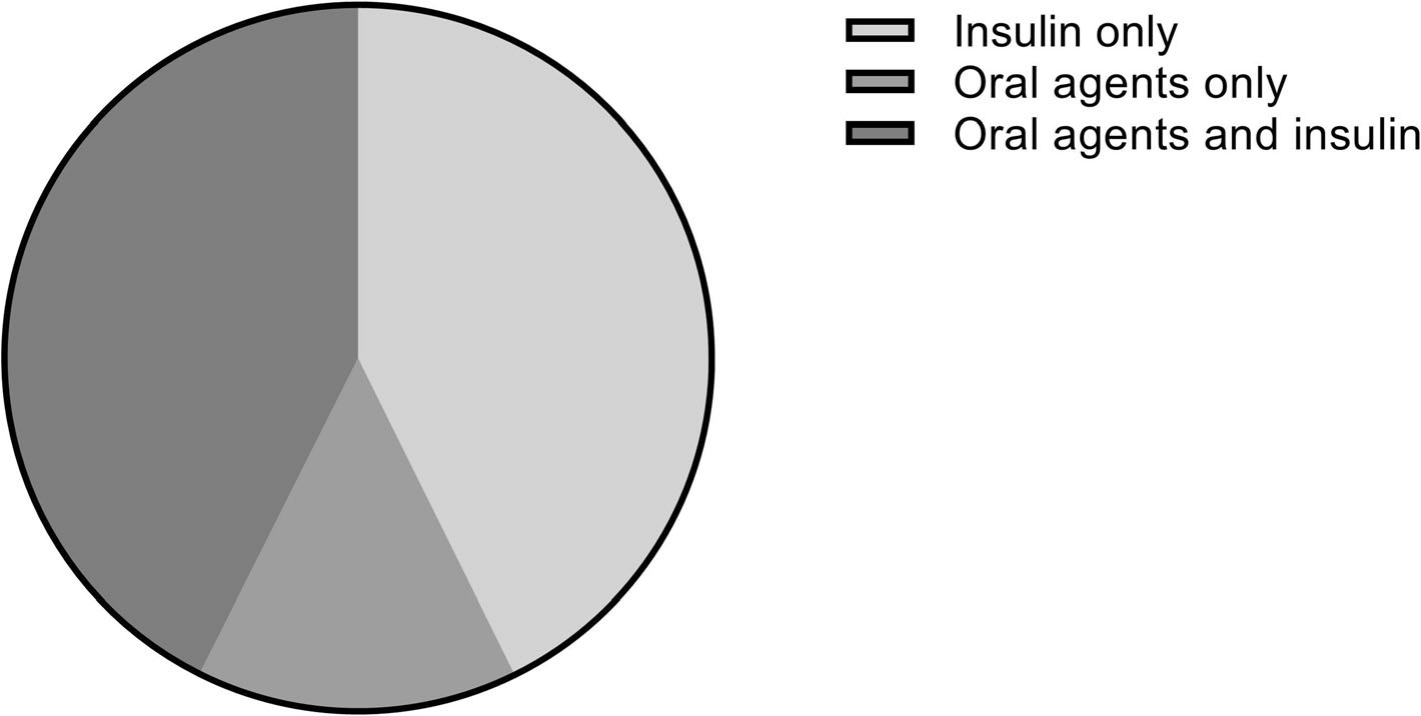
Treatment categories in all patients with reported treatment included in DPARD up to 2020. DPARD = Dutch Pediatric and Adult Registry of Diabetes

**Fig. 2:**
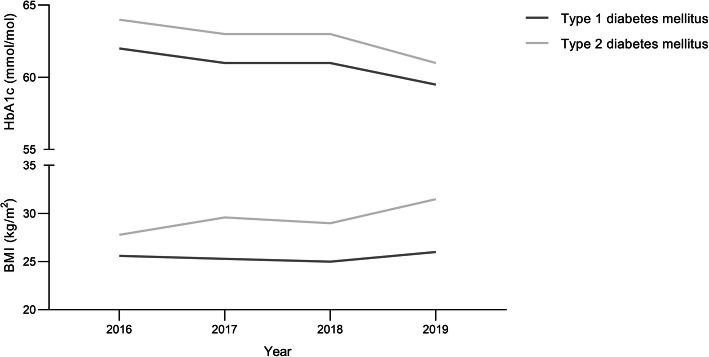
Trends in glycemic control and body mass index by diabetes type in patients included in DPARD. DPARD = Dutch Pediatric and Adult Registry of Diabetes, BMI = body mass index

Figure 1 shows treatment categories among patients included in DPARD. Most patients were treated with insulin (42.7%) or a combination of insulin and oral glucose-lowering drugs (42.6%). A minority used oral agents only (14.7%). Almost all patients with type 1 diabetes used insulin monotherapy (93.1%). In type 2 diabetes, the majority of the patients used a combination of insulin and oral glucose-lowering medication (59.2%). The proportion of the type 2 diabetes patients using insulin monotherapy (20.5%) or oral glucose-lowering agents was equal (20.3%). Figure 2 shows trends over time in glycaemic control expressed in HbA1c levels and body mass index by diabetes type. HbA1c levels decreased in patients with both diabetes types from 2016 up to January 2020. During this period, median HbA1c declined from 62 to 59.5 mmol/mol (7.8 to 7.6%) in type 1 and from 64 to 61 mmol/mol (8.0 to 7.7%) in type 2 diabetes patients. In contrast, BMI levels increased from 25.6 to 26.0 kg/m^2^ in type 1 and from 28.3 to 31.5 kg/m^2^ in patients with type 2 diabetes within the same timeframe. The extent to which parameters were rendered and covered, varied significantly. Data on blood pressure was delivered least frequently by hospitals, whereas age, sex, and HbA1c were provided by all healthcare centres. In each institution, data coverage on age and sex was complete. Structural follow-up of children with diabetes mellitus had taken place in two centres, comprising 9.7 and 29.3% of the outpatients with diabetes mellitus in those hospitals. Percentages of missing data in Tables 1 and 2 varied from 0% in age and sex to 100% in blood pressure and eGFR in children. Among variables on physical examination, height and weight were the variables with the lowest missing rates (both 29.7%). Of all laboratory parameters, HbA1c had the lowest missing data from with 0.3% missing among children. Blood pressure, eGFR and urinary albumin were variables with the highest missing rates, up to 61.3% in blood pressure. Data from patients with other or secondary causes of diabetes mellitus had the highest missing rates among all types of diabetes mellitus, including the unclassified patients.

Compared to type 2 diabetes patients, patients with type 1 diabetes mellitus had higher percentages of missing data on BMI, blood pressure, eGFR, urinary albumin and cholesterol.

Missing rates of diabetes duration and HbA1c were lower in patients with type 1 than in type 2 diabetes mellitus. Patients with unclassified diabetes mellitus had lower percentages of missing data than classified types of diabetes mellitus, except for diabetes duration and blood pressure. In 64.3% of adults, no information on diabetes treatment was given. The proportion of missing data on diabetes medication was relatively small in type 2 diabetes patients (26%) and large in patients with unclassified diabetes mellitus (91.4%). Missing data on diabetes medication was equal in men and women (64.0% vs. 64.7%). Data completeness of most variables improved over the years, with missing values from the variable HDL-cholesterol decreased the most from 96.7% in 2016 to 19.1% in 2019. The only variables with decreasing completeness of data were diabetes classification and diabetes duration (from 2.0 to 78.5% missing data, and from 0.9 to 55.5% missing data, respectively).

## Discussion

The objective of this study was to set forth the rationale, design and first results of the Dutch Pediatric and Adult Registry of Diabetes (DPARD), which aims to gather reliable information about prevalence, complications, comorbidity, treatment effects, and quality of care among all diabetes patients treated at outpatient clinics in secondary and tertiary care across the Netherlands. Of 20,087 patients included in DPARD, 18,714 have visited the outpatient clinic up until January 2020. DPARD harbours many clinical parameters providing information on the Dutch outpatient population with diabetes mellitus. Data are collected directly from electronic health records of local hospitals. Feedback on diabetes care is provided to participating hospitals in order to highlight areas in need of improvement. DPARD also renders valuable insight in how clinical parameters are captured from electronic health records.

In nearly half of patients included in DPARD, diabetes type was not provided. This classification issue has also been addressed in other large national clinical diabetes registries, [12, 32] and may be caused by misclassification, misdiagnosis and miscoding. Diagnosis classification and diabetes duration were the only variables showing a decrease of data completeness over time, caused by inclusion of a few large healthcare institutions not providing this information. However, baseline characteristics of unclassified patients are similar to the entire population in DPARD, suggesting that the unclassified part of our population has a comparable distribution of diabetes type. Other national diabetes registries use algorithms including age, treatment and eventually clinical diabetes classification to overcome non-classification, [32, 33] which often leads to discarding part of the data when presenting data [12]. Although unsatisfying, we feel that this lack of sufficient data on diabetes classification is crucial to share, as it sends an important signal to both local hospitals and national stakeholders to address this issue. Within the DPARD research group, a project will be soon started in two local hospitals to stimulate data completeness of diabetes classification in electronic health records.

Approximately 8% of the patients with known diabetes classification had other kinds of diabetes than type 1 or type 2. This is a heterogeneous group of patients consisting of monogenic syndromes (viz. neonatal diabetes mellitus and maturity-onset diabetes of the young), diseases of the exocrine pancreas, and drug-induced diabetes mellitus. Due to the diverse aspects of this group, age and diabetes duration are comparable to patients with type 1 diabetes, and BMI levels are corresponding with type 2 diabetes patients. Relatively many children were found in this group, which may be explained by monogenetic diabetes mellitus accounting for up to 4.2% of diabetes in children [34]. Remarkable are the low percentage of male patients in this group. In current literature, no data exist on sex distribution in this subgroup. Low HbA1c values may be explained by monogenetic diabetes mellitus, often showing relatively low HbA1c levels [35].

As expected, median age in patients with type 1 diabetes was much lower than in patients with type 2. In comparison, type 1 patients treated in specialist care in 2019 in the National Diabetes Registry (NDR) of Sweden were fairly older also when focusing on adults only, whereas type 2 diabetes patients were slightly younger, [36] which may be explained by different referral patterns.

The higher ages of type 1 diabetes patients in the NDR could be partly explained by the lack of children in this sample from the NDR. In the national diabetes registry from Scotland comprising both children and adults, mean age of type 1 diabetes patients was similar to those in DPARD [33].

Additionally, the Diabetes Collaborative Registry (DCR) in the USA showed a comparable age in patients with type 2 diabetes mellitus. Interestingly, the registries from Scotland and the USA both register primary care, while DPARD currently solely focuses on outpatient care.

HbA1c levels were slightly higher in type 2 diabetes compared to type 1. Data from other national diabetes registries showed HbA1c levels to be equal among diabetes types [36] or HbA1c levels higher in type 1 diabetes [37]. A possible explanation is the longer diabetes duration in type 2 diabetes patients than in type 1 diabetes patients due to referral from primary to secondary or tertiary care in the Netherlands, resulting in higher HbA1c levels. Furthermore, DPARD had higher HbA1c levels in comparison with the patients from the NDR treated in specialist care, which was also the case with type 2 diabetes patients in the DCR. Higher HbA1c values in DPARD could possibly be explained by the high percentage of tertiary care centres with complex patient populations currently included, diabetes duration, or ethnic composition of DPARD [38, 39].

In 1996, when the NDR was initiated, HbA1c values were more comparable to DPARD [36]. Since the foundation of the NDR, HbA1c levels of included patients declined with 8.5 and 7.5 mmol/mol (0.9 and 0.8%) in type 1 and type 2 diabetes patients, respectively. In DPARD, a similar yet smaller trend is visible. Although optimistic, whether this decline proceeds as it does in the NDR remains to be seen. Moreover, it is unclear whether lowering of HbA1c levels in the NDR is driven by data or caused by population trends, policy changes or improved treatment guidelines.

The primary analysis exposed the high occurrence of missing data. Electronic health records are a promising data source for public health, medical research and patients registries. Several national diabetes registries use electronic health records as data source, [40, 41] yet these records are not primarily designed for use by registries resulting in missing data. Data completeness is known to vary per variable and across registries, [42] depending on whether the measurement is performed, whether it is documented in the electronic health record, whether the recording is done in a structured and discrete fashion, and whether data are provided by hospitals in the right format. Since DPARD data are provided by the healthcare institutions via automatic data upload, selection of variables with favorable outcomes is unlikely. Therefore, no systematic differences are expected between patient records with missing data and those with complete data, thus not compromising data generalizability. Furthermore, data completeness is improving over the years, and is assumed to improve even more in the future as the current trend continues and the awareness of the benefits and potential of registries increases. Testing on at least one of the autoantibodies ICA, GAD or IA2 was recorded in 90.9% of the children and in 38.2% of the adults with type 1, auto antibody status of other patients is not known. We believe this is mostly due to the recording and delivering of data by the hospitals, since we requested but did not oblige hospitals to deliver data on autoantibodies, or that patients were diagnosed in another hospital or tested elsewhere, causing data on autoantibody status not to be discretely recorded in electronic health records. Surprisingly, blood pressure, eGFR and urinary albumin had the highest missing rates from the laboratory and physical examination parameters. Data completeness regarding blood pressure values in DPARD was comparable with other studies using data derived from electronic health records, ranging between 0.1 and 51% [43]. In national clinical diabetes registries worldwide, blood pressure, eGFR, and urinary albumin were also not consequently recorded in al registries, [44] yet these parameters give crucial information about the risk of vascular complications and these parameters may be recorded discretely in electronic health records and are embedded in national and international guidelines [22, 45].

Missing rates on those variables were even higher in type 1 diabetes than in type 2, while diabetic nephropathy is the leading cause of mortality in type 1 diabetes, [46] and the association between blood pressure in vascular outcomes is similar in type 1 and type 2 diabetes mellitus [47]. This emphasizes the need for improving complete data capture so that the registry would be more robust in yielding actionable information. Adequate recording of variables in electronic health care systems is currently being addressed on a national level in various projects. Automatic filling of variables in the DPARD registry based on pre-set criteria may also be promising to improve data completeness. One of the major electronic health record companies in the Netherlands has integrated DPARD in their electronic health record environment. Future ventures on this topic include combining forces with other registries such as the Dutch mortality and hospitalization registries as well as international registries.

DPARD is the first diabetes registry in the Netherlands aiming for national coverage. However, at this point nearly half of the patients in DPARD do not have a diabetes classification yet, which renders the comparison of features of patients defined as type1 and type 2 of limited value in these preliminary results. In addition, missing rates on several parameters are high. While a valuable registry both nationally and internationally, the need for improvement is well recognized and will be effectuated on short notice. Within 2 years, we expect to include all outpatient diabetes clinics across the Netherlands with data containing diabetes classification of every patient included and far less missing values across all data due to advancements in data scripts and resources both in hospitals and on a national level.

With a currently estimated count of 12% of the Dutch outpatient population with diabetes mellitus, DPARD provides a first glimpse into clinical characteristics, treatment and long-term outcomes all Dutch diabetes patients treated in outpatient clinics. This enables us to benchmark quality information between hospitals to identify areas in need of improvement in order to enhance diabetes care in the Netherlands, as well as allowing for comparison of diabetes care on an international level.

## Data Availability

The datasets generated and analysed during the current study are not publicly available because hospitals delivering data remain ownership of their data. Furthermore, DPARD-data contain information that could compromise research participant privacy but are available from the corresponding author on reasonable request. Data are available from the corresponding author on reasonable request.

## Abbreviations

BMI: Body Mass Index
DCR: Diabetes Collaborative Registry
DICA: Dutch Institute for Clinical Auditing
DPARD: Dutch Pediatric and Adult Registry of Diabetes
DVN: Dutch federation of diabetes patients
GAD: Anti-glutamic acid decarboxylase
ICHOM: International Consortium of Health Outcome Measurement
MRDM: Medical Research Data Management
NDR: National Diabetes Registry
NIV: Netherlands Association of Internal Medicine
NVK: Dutch Association for Paediatrics
IA2: Islet antigen 2
ICA: Islet cell antibodies
